# Effect of 24 Sessions of High-Intensity Aerobic Interval Training Carried out at Either High or Moderate Frequency, a Randomized Trial

**DOI:** 10.1371/journal.pone.0088375

**Published:** 2014-02-07

**Authors:** Håvard Hatle, Per Kristian Støbakk, Harald Edvard Mølmen, Eivind Brønstad, Arnt Erik Tjønna, Sigurd Steinshamn, Eirik Skogvoll, Ulrik Wisløff, Charlotte Björk Ingul, Øivind Rognmo

**Affiliations:** 1 K.G. Jebsen Centre of Exercise in Medicine, Norwegian University of Science and Technology (NTNU), Trondheim, Norway; 2 Department of Circulation and Medical Imaging, NTNU, Trondheim, Norway; 3 Department of Pulmonary Medicine, St Olav University Hospital, Trondheim, Norway; University of Bath, United Kingdom

## Abstract

**Purpose:**

The training response of an intensified period of high-intensity exercise is not clear. Therefore, we compared the cardiovascular adaptations of completing 24 high-intensity aerobic interval training sessions carried out for either three or eight weeks, respectively.

**Methods:**

Twenty-one healthy subjects (23.0±2.1 years, 10 females) completed 24 high-intensity training sessions throughout a time-period of either eight weeks (moderate frequency, MF) or three weeks (high frequency, HF) followed by a detraining period of nine weeks without any training. In both groups, maximal oxygen uptake (VO_2max_) was evaluated before training, at the 9^th^ and 17^th^ session and four days after the final 24^th^ training session. In the detraining phase VO_2max_ was evaluated after 12 days and thereafter every second week for eight weeks. Left ventricular echocardiography, carbon monoxide lung diffusion transfer factor, brachial artery flow mediated dilatation and vastus lateralis citrate maximal synthase activity was tested before and after training.

**Results:**

The cardiovascular adaptation after HF training was delayed compared to training with MF. Four days after ending training the HF group showed no improvement (+3.0%, p = 0.126), whereas the MF group reached their highest VO_2max_ with a 10.7% improvement (p<0.001: group difference p = 0.035). The HF group reached their highest VO_2max_ (6.1% increase, p = 0.026) twelve days into the detraining period, compared to a concomitant reduction to 7.9% of VO_2max_ (p<0.001) above baseline in the MF group (group difference p = 0.609).

**Conclusion:**

Both HF and MF training of high-intensity aerobic exercise improves VO_2max_. The cardiovascular adaptation following a HF programme of high-intensity exercise is however delayed compared to MF training.

**Trial Registration:**

ClinicalTrials.gov NCT00733941.

## Introduction

Maximal oxygen uptake (VO_2max_) is considered the best measure of aerobic capacity and an increase in VO_2max_ is the most common way of demonstrating an effect of aerobic exercise training [1]. The most efficient exercise intensity for improving VO_2max_ has been found to be close to maximal heart rate (HR_max_) in both healthy subjects [2] as well as in different patient groups [3]–[5]. Interval training is an effective way of performing high-intensity aerobic exercise, and our research group have previously demonstrated that high-intensity interval exercise at 90–95% of HR_max_, performed 2–3 times per week is superior to continuous moderate exercise at 60–70% of HR_max_ for improving VO_2max_ when total exercise volume is equalized [6].

The knowledge of how a short and intensified period of high-intensity exercise influences the progression of VO_2max_ is however less clear. Such block periodization could provide a highly time-efficient method for improving VO_2max_ within a very short time-period [7]. A recent study of Swiss alpine junior skiers found that 15 high-intensity exercise sessions at 90–95% of HR_max_ performed over 11 days improved VO_2max_ by 6% when measured seven days after completing training [8]. However, two earlier studies have reported a decrease in VO_2max_ and work performance after two weeks of highly intensified training in competitive cyclists [9], [10]. This indicates that the assessment of VO_2max_ may be dependent of when the measurements are taking place after completing a highly intensified training period. Also the cardiovascular and pulmonary adaptations have shown to be depressed immediately after intensified exercise [11], [12]. Therefore, the aim of the present study was to investigate the progression of aerobic adaptation in young, healthy individuals completing 24 interval training sessions carried out over either 3 weeks or 8 weeks, respectively. A secondary aim was to study the cardiovascular, pulmonary, endothelial and skeletal muscle adaptation induced by the two training programmes.

## Methods

### Participants

**Figure 1:**
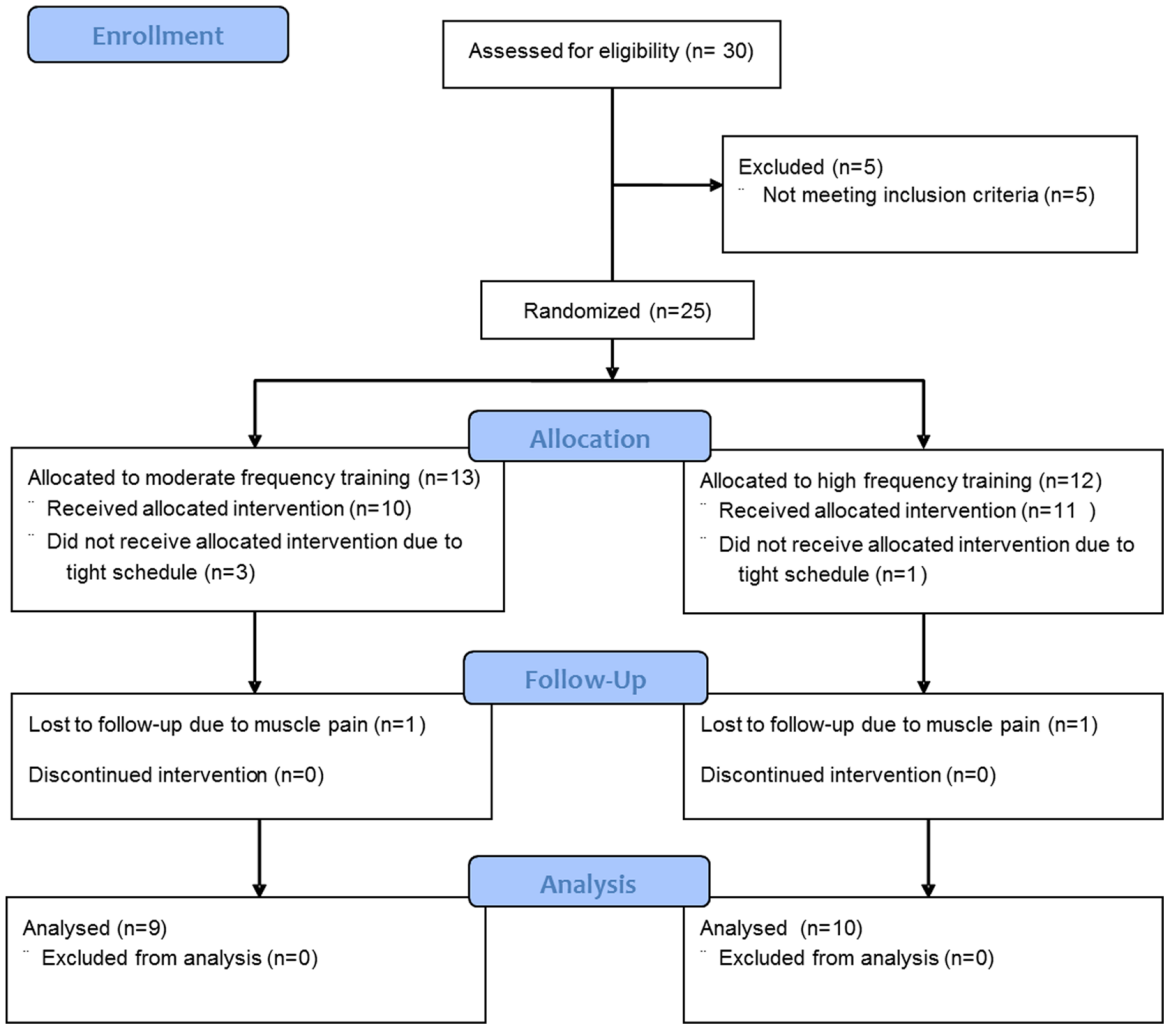
CONSORT flow diagram of the study design.

Twenty-one students (23.0±2.1 years, 10 females) were recruited from the Norwegian University of Science and Technology in Trondheim, Norway. Inclusion criteria were; healthy, non-smokers, BMI ≤27, age ≤30 years, not practising aerobic training more than twice per week and not exceeding a VO_2max_ of 50 and 60 ml · kg^−1^ · min^−1^ for women and men, respectively. The subjects were matched by gender and randomly assigned to a high frequency (HF) (n = 11; 6 females), or a moderate frequency (MF) (n = 10; 4 females) exercise group (Figure 1). The unit of Applied Clinical Research at the university carried out all randomization procedures to ensure complete blinded randomization. All participants signed written informed consent. The study protocol was approved by the regional committee for medical research ethics (REK midt 4.2008.1755) and registered by ClinicalTrials.gov (Identifier NCT00733941). The protocol for this trial and supporting CONSORT checklist are available as supporting information; see Checklist S1 and Protocol S1. The study was carried out from September 2008 to January 2009.

### Exercise Protocol

The training period consisted of 24 training sessions carried out over a time span of either eight or three weeks for the MF group and the HF group, respectively. A subsequent detraining period consisted of nine weeks without aerobic training, except necessary daily activities. Both groups performed high-intensity aerobic training running uphill on a treadmill, with individually adjusted speed and inclination, supervised by a personal trainer. Each training session consisted of 10 minutes warm-up followed by 4×4-minute intervals at 90–95% of HR_max_, alternating with 3-minute of active recovery at 70% of HR_max_, ending with a 5-minute cool-down, giving a total exercise time of 40 minutes per session. Heart rate (HR) was monitored by HR recorders (Polar, Finland) to ensure that all training sessions was carried out at definitive intensities.

### Maximal Oxygen Uptake Testing

**Figure 2:**
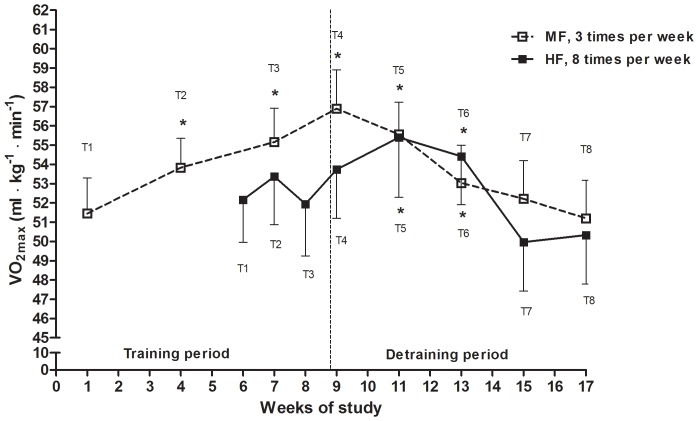
Mean values and standard error of the mean of VO_2max_ for the two groups during the training and the detraining period are shown. Vertical dotted line states the last day of training. Confer Table 2 for statistics and exact values at test points (T1–T8). * Indicates significant differences from baseline within groups.

Maximal oxygen uptake was tested using an individualized graded step incremental test on a treadmill (Woodway, PPS 55). After a 10 minutes warm-up, a facemask was placed on the subjects for metabolic measurements using a Metamax II (Cortex, Leipzig, Germany), where incline was constant at 10% and speed was increased by 1 km · h^−1^ every minute. Tests were terminated when candidates reached a VO_2_ plateau that remained stable despite increased work load, i.e. VO_2_ did not increase more than 2 mL · kg^−1^ · min^−1^ despite increased work load, and a respiratory exchange ratio (RER) >1.10. VO_2max_ was measured before study started (pre-test) and four days after the 24^th^ and final training session (post-test). VO_2max_ was also tested during the training period, i.e. at the 9^th^ and 17^th^ training session, and after these tests the subjects performed 3×4-minute intervals. In the detraining phase the subjects were tested 12 days after the last training session and thereafter every second week until the end of the eight week detraining phase. Thus, a total of eight VO_2max_-tests were conducted for both groups (Figure 2).

### Echocardiographic Measurements

Echocardiography at rest was performed at baseline and repeated six days after the training period ended. Measurements were also repeated after the nine weeks of detraining. Complete echocardiographic examinations were performed using a Vivid 7 scanner (GE Vingmed Ultrasound, Horten, Norway) with a phased-array transducer (3MS probe). Three cine loops from the three standard apical planes (four-chamber, two-chamber and long-axis) as well as short-axis (basal, mid-papillary and apical) were recorded in gray scale harmonic mode and tissue Doppler mode. B-mode recordings were done with an average of 53 frames per second and tissue Doppler with 154 frames per second. Measurements and calculations obtained were in accordance to standard procedures recommended by the American Society of Echocardiography. LV volumes and ejection fraction (EF) were calculated from apical recordings by modified biplane Simpson’s method. Inflow velocity of the left ventricle was measured by pulsed Doppler with the sample volume at the tips level of mitral valves. Measurements included early diastolic filling (E) and late diastolic filling (A). Stroke volume (SV) and cardiac output (CO) was calculated by Doppler flow measurement in the left ventricular outflow tract (LVOT). Tissue Doppler recordings of the mitral annulus were obtained from the septal and lateral points in four-chamber and in the anterior and inferior points in two-chamber views. Peak annulus velocity in systole (S’), early diastole (e’) and late diastole (A’) were measured as average of the four points.

### Lung Function

Lung function testing was performed before and four days after the training period. Flow-volume spirometry [13] was performed with the Master Screen Pneumo Spirometer (Jaeger, GmbH & Co, KG). The spirometry was calibrated daily, and the highest of two measurements with <5% variation was recorded [14]. Transfer Factor of the Lung for Carbon Monoxide (TLCO) was measured by the “single breath”-method with gas mixture of 10% helium, 0.3% carbon monoxide, 21% oxygen and 68.7% nitrogen [13] with the Master Screen Diffusion (Jaeger, GmbH & Co, KG). All lung function tests were performed at sea level at room temperature (20–22°C).

### Endothelial Function

Endothelial function was measured before and after the training phase. The measurements were done 1–3 weeks prior to study start and 10 days after the last training session for both groups. Endothelial function was measured as flow-mediated dilatation (FMD) with high-resolution vascular ultrasound using a 14 MHz echo Doppler probe (Vivid 7 System, GE Vingmed Ultrasound, Horten, Norway) according to guidelines [15]. The procedures for measuring FMD and blood pressure were recently published by our group [16]. All ultrasound images were analyzed in random order with EchoPACtm (GE Vingmed Ultrasound AS) by a blinded investigator.

### Muscle Biopsies and Citrate Synthase Activity

Muscle biopsies were taken at baseline and five days after completing the training programme. The biopsies were obtained from the vastus lateralis with a sterile 5 mm diameter biopsy needle (Pelomi, Denmark) under local anaesthesia. All samples were snap-freezed in liquid nitrogen immediately after sampling. For citrate synthase (CS) maximal activity measurements, the muscle samples were homogenized in CelLytic buffer (Sigma-Aldrich, USA) at 6000 shakes/min for 2×8 seconds in a Precellys24 tissue homogenizer (Bertin, France). The homogenate was then centrifugated at 10000 g for 10 min at 4°C and supernatant tested for CS activity. The CS maximal activity was determined by the use of citrate synthase Assay Kit (CS 0720, Sigma-Aldrich). Absorbance at 412 nm was measured on a Fluostar Omega spectrometer (BMG Labtech, Germany). Specific activity was calculated by dividing measured activity on the muscle extract protein concentration. Protein concentrations were determined by the method of Bradford [17].

### Statistical Analysis

Samples size was estimated for VO_2max_ as primary outcome measure using IBM SPSS SamplePower. The principal outcome variable VO_2max_ were analysed between groups using a linear model with correction for baseline value (ANCOVA) [18], and within groups comparisons were done using paired samples T-test. Values are expressed as mean and standard deviation (SD). A two-tailed significance-level of p<0.05 was acknowledged as statistical significant. All analyses were done by PASW 17.0 (SPSS Inc, Chicago). At five occasions a subject was unavailable for testing. Because of the Gaussian behaviour of the data and the low number of missing values, these time points were estimated by an imputed value using Missing Value Analysis (PASW 17.0) to complete the dataset [19].

## Results

### Subject Characteristics

There were no differences between the groups with respect to age, body weight, height and body mass index (BMI) at baseline (Table 1). One person in each group withdrew from the study reporting tight calf muscles, and a total of 19 subjects completed the training- and detraining phases and were included in the analysis (Figure 1). All subjects that completed the study fulfilled >95% compliance to the prescribed training programme.

**Table 1:** Group Characteristics.

	MF group (n = 9)	HF group (n = 10)	p-value
Age (yrs)	23.1±2.3	23.7±2.1	= 0.560
Weight (kg)	70.2±16.8	67.7±8.9	= 0.684
Height (cm)	175.0±11.3	173.0±8.7	= 0.709
BMI (kg/m^2^)	22.7±3.1	22.6±2.3	= 0.938

### Adaptation of VO_2max_

There was no baseline difference in VO_2max_ between the groups before the study started (Table 2). The MF group improved VO_2max_ by 4.6% (p = 0.036) already after eight training sessions, whereas no significant improvement was seen in the HF group at the same time point (+2.3%, p = 0.128; between group difference p = 0.449). After finishing 16 of the 24 sessions, we found an improvement in VO_2max_ of 7.1% (p = 0.002) in the MF group compared no improvement (−0.5% vs. baseline, p<0.128) in the HF group (between group difference p = 0.036) (Figure 2 and Table 2). At post-test, i.e. four days after ending training, the MF group reached their highest achieved VO_2max_ by 10.7% (p<0.001) above baseline, whereas no improvement (+3.0%, p = 0.126) was seen in the HF group (between group difference, p = 0.035). Twelve days into the detraining period (week 11 of the study) the HF group reached their highest VO_2max_ with a value of 6.1% (p = 0.026) above baseline, compared to a VO_2max_ of 7.9% (p<0.001) above baseline in the MF group at the same time point (group difference p = 0.609) (Figure 2). When considering the individual subjects’ highest VO_2max_ (Highest-VO_2max_), irrespective of which VO_2max_-test it was reached, the MF and HF groups improved their VO_2max_ with 12.1% (p<0,001) and 7.5% (p = 0.010), respectively (between group difference, p = 0.319) (Table 2).

**Table 2:** Values of VO_2max_ expressed as means ± SD for both groups during the training and detraining periods.

	MF group		HF group		Between gr.
Test points	VO_2max_	p-value	VO_2max_	p-value	p-value
**1** (pre-test)	51.5±5.5	–	52.2±7.0	–	= 0.290
**2** (after 8 sessions)	**53.8±4.6**	** = 0.036**	53.4±7.9	= 0.128	= 0.449
**3** (after 16 sessions)	**55.2±5.3**	** = 0.002**	51.9±8.5	= 0.829	** = 0.036**
**4** (post-test, 24 sessions)	**56.9±6.1**	**<0.001**	53.7±8.0	= 0.126	** = 0.035**
**5** (12 d post exercise)	**55.6±5.0**	**<0.001**	**55.4±9.8**	** = 0.026**	= 0.609
**6** (4 wk post exercise)	**53.0±5.9**	** = 0.029**	**54.4±7.9**	** = 0.018**	= 0.798
**7** (6 wk post exercise)	52.2±5.9	= 0.339	50.0±8.1	= 0.064	= 0.115
**8** (post-detraining)	51.2±5.9	= 0.758	50.3±8.1	= 0.118	= 0.493
Highest-VO_2max_	**57.5±5.2**	**<0.001**	**56.0±9.7**	** = 0.010**	= 0.319

Post-test refers to values at the first test after training, which was four days after last exercise session. Highest-VO_2max_ refers to collected highest measured value of subjects within each group, independent of at which test point after training it occurred. VO_2max_ is presented as ml · kg^−1^ · min^−1^. Significant differences from baseline within groups and differences between groups are typed in bold. Between group differences are post-test values.

After 4 weeks of detraining (week 13 into the study, Figure 2 and Table 2) the VO_2max_ in the MF group was still elevated compared to baseline (+2.9%, p = 0.029), but was reduced compared to their highest achieved time point (i.e. 4 day post exercise: p = 0.003). The VO_2max_ in the HF group was after 4 weeks of detraining 4.2% above baseline (p = 0.018) and not reduced relative to the group’s highest measurement (i.e. 12 d post exercise: p = 0.343) (Figure 2 and Table 2). After 6 weeks of detraining (week 15 into the study) VO_2max_ was back to baseline level in both groups, i.e. +1.5% compared to baseline in the MF group, (p = 0.339) and −4.2% in the HF group (p = 0.064).

### Left Ventricular Adaptations

Stroke volume increased by 14.5% after the training period in the MF group (p = 0.028), while no change was observed in the HF group (p = 0.369) (Table 3). Left ventricular ejection fraction (EF) increased in the MF group (12.1%, p = 0.050), whereas it decreased by 11% (p<0.001) after HF training, but it is notable that EF was 15% higher in the HF group compared to MF group at baseline (between group difference, p = 0.050). End diastolic volume (EDV) increased only in the HF group, by 10.1% (p = 0.050). S’ tended to increase in the MF group (7.8%, p = 0.056), but no change was observed in the HF group (between group differences: p = 0.490). E, A and e’ did not change in any group after the training period.

**Table 3:** Parameters of heart function for both groups before and after training and after detraining.

	MF group					HF group				
	Pre-test	Post-test	p-value	Detraining	p-value	Pre-test	Post-test	p-value	Detraining	p-value
**HR_rest_,** b/min	58±6	58±9	= 0.997	59±9	= 0.901	65±9	57±8	= 0.034	66±12	= 0.954
**BP_sys_,** mmHg	117.0±10.0	118.0±11.0	= 0.943	108.0±9.0	= 0.025	118.5±10.5	123.0±9.0	= 0.343	121.5±16	= 0.561
**BP_dia_,** mmHg	74.0±6.0	74.0±7.0	= 0.992	68.0±3.0	= 0.074	74.0±8.5	74.0±5.0	= 0.784	71.0±9.5	= 0.454
**EDV,** ml	120.0±28.0	129.0±40.0	= 0.176	118.6±23.0	= 0.408	119.5±20.0	131.5±25.0	= 0.034	131.0±20.0	= 0.024
**SV,** ml	77.0±21.0	88.0±18.0	= 0.028	87.0±21.0	= 0.192	85.0±14.1	89.0±18.5	= 0.369	93.5±11.0	= 0.031
**S’,** cm/s	8.95±8.1	9.65±7.5	= 0.056	9.05±10.5	= 0.096	9.50±16.1	9.60±14.3	= 0.813	9.70±14.6	= 0.507
**EF,** %	58.0±5.8	65.0±4.3	= 0.050*	63.5±5.9	= 0.380	66.5±5.1	59.0±2.7	<0.001	66.0±5.2	= 0.805
**E/A**	2.4±0.7	2.7±0.9	= 0.239	2.4±0.7	= 0.819	2.3±0.8	2.8±0.6	= 0.070	2.3±0.7	= 0.790
**e’**	15.9±2.2	16.1±2.4	= 0.741	16.0±1.8	= 0.524	15.6±2.6	15.7±1.9	= 0.862	15.7±2.1	= 0.734

Values are expressed as means ± SD. HR_rest_; resting heart rate, BP_sys_; systolic blood pressure, BP_dia_; diastolic blood pressure, EDV; end diastolic volume, SV; stroke volume, S’; peak annulus velocity in systole, e’; early diastole, and A’; late diastole were measured as average of the four measures. EF; ejection fraction. P-values are within-groups differences from pre- to post-test, and between group differences for post-test values.

* Significant differences between groups (p<0.05).

During the detraining period SV and EF returned to baseline values in the MF group. In the HF group SV increased by 10.0% (p = 0.031) during the detraining, and EF and S’ returned to baseline values.

### Lung Function

Flow volume spirometry values did not change at any point of measurement in either group. TLCO was reduced by 11.3% (p = 0.004) in the HF group after the training period, while the MF group increased diffusion capacity by 6.0% (p = 0.041) (Table 4). Haemoglobin levels did not differ during the study period, and thus TLCO was not corrected for haemoglobin levels.

**Table 4:** Physiological variables for both groups before and after the training period.

	MF group			HF group			Between gr. p-value
	Pre-test	Post-test	p-value	Pre-test	Post-test	p-value	
**FMD,** %	7.4±3.6	7.5±2.5	= 0.952	7.5±2.0	7.6±2.8	= 0.910	= 0.948
**TLCO,** conductance units	9.36±1.16	9.92±1.48	= 0.041	10.61±2.14	9.40±1.79	= 0.004	= 0.094
**CS,** µmole/ml/min/mg protein	0.38±0.10	0.52±0.09	<0.001	0.30±0.12	0.37±0.12	= 0.185	= 0.009

Values are expressed as means ± SD. FMD; Brachial artery flow mediated dilatation, CS; citrate synthase activity, TLCO; transfer factor of the lung for carbon monoxide. Between group differences are post-test values. P-values are within-groups differences from pre- to post-test, and between group differences for post-test values.

### Endothelial Function

Flow mediated dilation was unchanged from before to after training both within the MF group (7.4±3.6 vs. 7.5±2.5% (p = 0.952) and within the HF group (7.5±2.0 vs. 7.6±2.8% (p = 0.910) (between group difference p = 0.943) (Table 4).

### Citrate Synthase Activity

The vastus lateralis citrate synthase maximal activity relative to the muscle extract protein concentration was increased by 39% in the MF group (from 0.38±0.10 to 0.52±0.10 µmol · ml^−1^ · min^−1^ · mg^−1^ protein, p<0.001) (Table 4). We also found a 25% increase in activity in the HF group (from 0.30±0.12 to 0.37±0.11 µmol · ml^−1^ · min^−1^ ·mg^−1^ protein), but this improvement did not reach statistical significance (p = 0.185; between group difference p = 0.009).

## Discussion

The main finding of this study was that both HF and MF training of high-intensity aerobic exercise improves VO_2max_. We found however no improvements in VO_2max_ immediately after ending the HF training programme. In addition, we found a reduction in lung diffusion capacity and indications of a reduction in cardiac function in HF immediately after training. This indicates a possibility of fatigue when exposed to such a high exercise dose. However, during the detraining period there was a delayed increase in VO_2max_ in the HF group demonstrating the necessity of sufficient rest to adapt from such a heavy exercise training programme. The MF training programme, in contrast, had a positive effect for improving the aerobic capacity in a progressive manner throughout the training period.

### Maximal Oxygen Uptake

Previous studies have demonstrated that VO_2max_ improves after HF aerobic training [20], [21], suggesting a possibility to improve VO_2max_ in reduced time compared to more moderate training regimes. In line with this, a recent study by Breil et al. [8] involving Swiss alpine skiers, showed that it is possible to boost the aerobic capacity by means of a 6% increase of VO_2max_ using a high-intensity interval training “shock” of 15 sessions in 11 days. In our study we found a quite different progression of the adaptation between MF- and HF-training, and the highest VO_2max_-values occurred at different time points. In the MF group the adaptation to physical training progressed in a regular manner. VO_2max_ increased progressively in the MF group during the training period, and reached highest value at post-test, four days after ending training period (+10.7%, p<0.001). Contrary, in the HF group there was no change in VO_2max_ during the training period. In fact, we measured a small, but not significant reduction at the VO_2max_-test performed at the 16^th^ training session, 14 days into the training regime. Furthermore, the HF group showed no improvements four days after ending 24 sessions of training (+2.9%, p = 0.126), and improved their VO_2max_ first after another week of detraining (+6.1%, p = 0.026), i.e. 12 days after finishing training. Interestingly, despite training for only 3 weeks the HF group still required 5 weeks to reach their highest measured VO_2max_ (+6.1% from baseline) whereas the MF group reached an improvement of 7.1% immediately after completing their 6 weeks of moderate training.

Our results show that the highly intensified training load experienced by the HF group was too severe to progressively adapt compared to the MF training programme. It is assumed that recovery processes continue through homoeostatic restoration of the body after training until a compensation effect may occur [22]. The most common way to adapt and improve physical fitness after training is to recover sufficiently and then perform the next exercise session at the peak of the compensation phase [23]. However, It has been described that athletes may experience acute feelings of fatigue and decrease in performance following periods of intensified training [24]. Intensified training can thus result in a temporarily decline in performance, but when sufficient periods of recovery are provided, a supercompensation may occur with enhanced performance compared to baseline levels [24]. As it is possible to recover within a 2-week period [25], it may be argued that this is a relatively normal condition and thus a normal part of a strenuous training process. The lack of improvement of VO_2max_ in the HF group during the training period, along with the compensation effect in the detraining phase, suggests that too severe training load was induced, as opposed to the progressive improvement of VO_2max_ in the MF group suggesting sufficient rest between the exercise sessions. However, it is still possible that more well-trained athletes could have acquired greater adaptation to HF training compared with our moderately trained students. There was however only one drop-out in each group, despite the higher physical demands in the HF group. The high compliance rate might be due to the young healthy population and a strong will to complete the training programme.

After 6 weeks of detraining, that started after the last exercise session, VO_2max_ returned to baseline values both in the MF and HF group. Mujika and Padilla [26] found that recently trained subjects most often reverse their gained VO_2max_ when detraining extends 4 weeks [27], whereas Pedersen et al. [28] found that the inactivity period simply reverses the training effect in the same manner as it emerged. Coyle et al. found that the decline in VO_2max_ stabilized after 8 weeks of detraining [29]. Although VO_2max_ in both groups in our study were still elevated compared to baseline VO_2max_ at 4 weeks into the de-training period, our findings suggest an earlier reduction of VO_2max_ relative to highest measured value in the MF group, i.e. after 4 weeks compared to 6 weeks in the HF group. The reason for this may simply correspond to the latency of their highest VO_2max_ value in the HF group, which occurred 2 weeks after training cessation. The gained aerobic capacity in both groups thus declined in a similar pattern from the highest reached value, being back at baseline 4 weeks after their highest measurement.

### Cardiovascular Response

We observed that stroke volume increased significantly in the MF group in response to the interval training in line with other studies [2], [5], [30]. In the HF group we found an increased end diastolic volume. In the HF group EF was reduced, suggesting a depressed left ventricular adaptation after the training period. The reduction in the systolic function is in concordance with another study that revealed a negative myocardial effect immediately after a strenuous exercise programme [12]. It should be emphasized that heart rate was reduced in the HF group after the exercise period, which may have affected the cardiac measurements. The cardiac measurements were secondary endpoints and the study is thus too small to draw firm conclusion. As expected, the diastolic measurements at rest did not improve, confirming existing literature that young healthy sedentary subjects on beforehand have a normal and optimal diastolic function [31].

During detraining in the MF group we observed that the stroke volume and the EF returned to baseline values. This indicates loss of the training induced adaptations caused by inactivity. The groups however differed in their adaptations during detraining. Stroke volume increased during detraining amongst the HF group and this was attributable to the increased end diastolic volume. EF was subsequently normalized. This indicates that recovery is crucial to achieve cardiac improvements after a strenuous physical activity programme. However, although the HF group increased stroke volume during detraining, the inactivity period returned VO_2max_ to baseline values.

It has previously been suggested that intense exercise may induce stress that impairs the systemic endothelial function [32]. We found however no signs of endothelial dysfunction as both groups showed the same FMD of the brachial artery both before and after training.

### Lung Function

The MF group increased pulmonary diffusion capacity after the training period. Diffusion capacity may have increased in relation to the increased stroke volume seen in the MF group.

### Peripheral Adaptations

The mitochondrial citrate synthase maximal activity improved more in the MF group compared to the HF group after the training programmes. Our results are in accordance with previous high-intensity exercise training studies showing that maximal activity of the citrate synthase is normally upregulated by 15–35% after strenuous training [33]. In line with our results, a recent study concluded that in skeletal muscle of trained individuals, the maximal activity of citrate synthase were enhanced to a greater extent by having a day of rest between the exercise sessions compared with training daily for 3 weeks [34].

### Study Strengths and Limitations

This study is small with few subjects in each group. However, the high number of test points throughout the study with a steady incline and decline of VO_2max_ in the MF group, is indicating that changes not just being measurement variations. Also, the VO_2max_-tests consistently showed plateaus of the O_2_-curve and high RER-values above 1.10, suggesting maximal effort during all tests. As it is difficult to predict when each subject achieves their highest VO_2max_ value after a training programme, an even more frequent test programme could have revealed even higher maximum values. More frequent testing could on the other hand elicit an unwanted training effect in the detraining period. Another aspect would have been to elucidate which mechanisms were causing the signs of fatigue during and after HF training. However, this study was not designed to examine these underlying mechanisms. The study was originally designed also to investigate retraining after the detraining period, but due to the strain of the participants, this part of the study was not implemented.

## Conclusions

It has previously been shown that it is possible to boost VO_2max_ by a short and intensified high-intensity interval training period. In our study we found a different progression of the adaptation between MF- and HF-training, and the highest VO_2max_-values occurred at different time points. The adaptation following a HF programme of high-intensity exercise was delayed compared to training with MF. The HF programme may induce an initially fatigue which may limit the cardiopulmonary function whereas the latter programme follows a more progressive adaptation.

## Supporting Information

Protocol S1 **Trial protocol.**
(DOC)

Checklist S1 **CONSORT checklist.**
(DOC)

## References

[1] Bassett DR Jr, Howley ET (2000) Limiting factors for maximum oxygen uptake and determinants of endurance performance. Med Sci Sports Exerc 32: 70–84. 10647532 10.1097/00005768-200001000-00012

[2] Helgerud J, Hoydal K, Wang E, Karlsen T, Berg P, et al (2007) Aerobic high-intensity intervals improve VO2max more than moderate training. Med Sci Sports Exerc 39: 665–671. 17414804 10.1249/mss.0b013e3180304570

[3] Rognmo O, Hetland E, Helgerud J, Hoff J, Slordahl SA (2004) High intensity aerobic interval exercise is superior to moderate intensity exercise for increasing aerobic capacity in patients with coronary artery disease. Eur J Cardiovasc Prev Rehabil 11: 216–222. 15179103 10.1097/01.hjr.0000131677.96762.0c

[4] Tjonna AE, Lee SJ, Rognmo O, Stolen TO, Bye A, et al (2008) Aerobic interval training versus continuous moderate exercise as a treatment for the metabolic syndrome: a pilot study. Circulation 118: 346–354. 18606913 10.1161/CIRCULATIONAHA.108.772822 PMC2777731

[5] Wisloff U, Stoylen A, Loennechen JP, Bruvold M, Rognmo O, et al (2007) Superior cardiovascular effect of aerobic interval training versus moderate continuous training in heart failure patients: a randomized study. Circulation 115: 3086–3094. 17548726 10.1161/CIRCULATIONAHA.106.675041

[6] Kemi OJ, Wisloff U (2010) High-intensity aerobic exercise training improves the heart in health and disease. J Cardiopulm Rehabil Prev 30: 2–11. 20040880 10.1097/HCR.0b013e3181c56b89

[7] Laursen PB, Jenkins DG (2002) The scientific basis for high-intensity interval training: optimising training programmes and maximising performance in highly trained endurance athletes. Sports Med 32: 53–73. 11772161 10.2165/00007256-200232010-00003

[8] Breil FA, Weber SN, Koller S, Hoppeler H, Vogt M (2010) Block training periodization in alpine skiing: effects of 11-day HIT on VO(2max) and performance. Eur J Appl Physiol. 10.1007/s00421-010-1455-1 20364385

[9] Jeukendrup AE, Hesselink MK, Snyder AC, Kuipers H, Keizer HA (1992) Physiological changes in male competitive cyclists after two weeks of intensified training. Int J Sports Med 13: 534–541. 1459749 10.1055/s-2007-1021312

[10] Snyder AC, Kuipers H, Cheng B, Servais R, Fransen E (1995) Overtraining following intensified training with normal muscle glycogen. Med Sci Sports Exerc 27: 1063–1070. 7564974 10.1249/00005768-199507000-00016

[11] McKenzie DC, O'Hare TJ, Mayo J (2005) The effect of sustained heavy exercise on the development of pulmonary edema in trained male cyclists. Respiratory Physiology & Neurobiology 145: 209–218. 15705536 10.1016/j.resp.2004.06.010

[12] Aslani A, Babaee Bigi MA, Moaref AR (2009) Effect of extreme exercise on myocardial function as assessed by tissue Doppler imaging. Echocardiography 26: 1036–1040. 19558519 10.1111/j.1540-8175.2009.00919.x

[13] Macintyre N, Crapo RO, Viegi G, Johnson DC, van der Grinten CP, et al (2005) Standardisation of the single-breath determination of carbon monoxide uptake in the lung. Eur Respir J 26: 720–735. 16204605 10.1183/09031936.05.00034905

[14] Miller MR, Hankinson J, Brusasco V, Burgos F, Casaburi R, et al (2005) Standardisation of spirometry. Eur Respir J 26: 319–338. 16055882 10.1183/09031936.05.00034805

[15] Pyke KE, Tschakovsky ME (2005) The relationship between shear stress and flow-mediated dilatation: implications for the assessment of endothelial function. J Physiol 568: 357–369. 16051630 10.1113/jphysiol.2005.089755 PMC1474741

[16] Tyldum GA, Schjerve IE, Tjonna AE, Kirkeby-Garstad I, Stolen TO, et al (2009) Endothelial dysfunction induced by post-prandial lipemia: complete protection afforded by high-intensity aerobic interval exercise. J Am Coll Cardiol 53: 200–206. 19130989 10.1016/j.jacc.2008.09.033 PMC2650775

[17] Bradford MM (1976) A rapid and sensitive method for the quantitation of microgram quantities of protein utilizing the principle of protein-dye binding. Anal Biochem 72: 248–254. 942051 10.1016/0003-2697(76)90527-3

[18] Vickers AJ, Altman DG (2001) Statistics notes: Analysing controlled trials with baseline and follow up measurements. BMJ 323: 1123–1124. 11701584 10.1136/bmj.323.7321.1123 PMC1121605

[19] Sterne JA, White IR, Carlin JB, Spratt M, Royston P, et al (2009) Multiple imputation for missing data in epidemiological and clinical research: potential and pitfalls. BMJ 338: b2393. 19564179 10.1136/bmj.b2393 PMC2714692

[20] Pollock ML, Miller HS, Linnerud AC, Cooper KH (1975) Frequency of training as a determinant for improvement in cardiovascular function and body composition of middle-aged men. Arch Phys Med Rehabil 56: 141–145. 1119922

[21] Gettman LR, Pollock ML, Durstine JL, Ward A, Ayres J, et al (1976) Physiological responses of men to 1, 3, and 5 day per week training programs. Res Q 47: 638–646. 1070734

[22] Viru A (1994) Molecular cellular mechanisms of training effects. J Sports Med Phys Fitness 34: 309–322. 7643575

[23] Kuipers H (1998) Training and overtraining: an introduction. Med Sci Sports Exerc 30: 1137–1139. 9662685 10.1097/00005768-199807000-00018

[24] Halson SL, Jeukendrup AE (2004) Does overtraining exist? An analysis of overreaching and overtraining research. Sports Med 34: 967–981. 15571428 10.2165/00007256-200434140-00003

[25] Halson SL, Bridge MW, Meeusen R, Busschaert B, Gleeson M, et al (2002) Time course of performance changes and fatigue markers during intensified training in trained cyclists. J Appl Physiol 93: 947–956. 12183490 10.1152/japplphysiol.01164.2001

[26] Mujika I, Padilla S (2000) Detraining: loss of training-induced physiological and performance adaptations. Part I: short term insufficient training stimulus. Sports Med 30: 79–87. 10966148 10.2165/00007256-200030020-00002

[27] Mujika I, Padilla S (2001) Cardiorespiratory and metabolic characteristics of detraining in humans. Med Sci Sports Exerc 33: 413–421. 11252068 10.1097/00005768-200103000-00013

[28] Pedersen PK, Jorgensen K (1978) Maximal oxygen uptake in young women with training, inactivity, and retraining. Med Sci Sports 10: 233–237. 750839

[29] Coyle EF, Martin WH 3rd, Sinacore DR, Joyner MJ, Hagberg JM, et al (1984) Time course of loss of adaptations after stopping prolonged intense endurance training. J Appl Physiol 57: 1857–1864. 6511559 10.1152/jappl.1984.57.6.1857

[30] Amundsen BH, Rognmo O, Hatlen-Rebhan G, Slordahl SA (2008) High-intensity aerobic exercise improves diastolic function in coronary artery disease. Scand Cardiovasc J 42: 110–117. 18365893 10.1080/14017430701744477

[31] George KP, Naylor LH, Whyte GP, Shave RE, Oxborough D, et al. (2009) Diastolic function in healthy humans: non-invasive assessment and the impact of acute and chronic exercise. Eur J Appl Physiol. 10.1007/s00421-009-1233-0 19820962

[32] Goto C, Higashi Y, Kimura M, Noma K, Hara K, et al (2003) Effect of different intensities of exercise on endothelium-dependent vasodilation in humans: role of endothelium-dependent nitric oxide and oxidative stress. Circulation 108: 530–535. 12874192 10.1161/01.CIR.0000080893.55729.28

[33] Gibala MJ, McGee SL (2008) Metabolic adaptations to short-term high-intensity interval training: a little pain for a lot of gain? Exerc Sport Sci Rev 36: 58–63. 18362686 10.1097/JES.0b013e318168ec1f

[34] Yeo WK, Paton CD, Garnham AP, Burke LM, Carey AL, et al (2008) Skeletal muscle adaptation and performance responses to once a day versus twice every second day endurance training regimens. J Appl Physiol 105: 1462–1470. 18772325 10.1152/japplphysiol.90882.2008

