# Supplementary material for: Effect of 24 Sessions of High-Intensity Aerobic Interval Training Carried out at Either High or Moderate Frequency, a Randomized Trial
Source: PLoS One. 2014 Feb 7;9(2):e88375. doi: 10.1371/journal.pone.0088375 (PMC3917911; doi:10.1371/journal.pone.0088375)
Supplement: Protocol S1 — Trial protocol. (DOC) [file pone.0088375.s001.doc]

**Protocol**

**Interval Training: Training, Detraining and retraining, an intervention study**

(The original protocol consisted of Training, detraining and retraining, but we did not conduct the final retraining part. This is a translation of the original Norwegian protocol. The translation was made just before submission of the manuscript).

**Background**

Physical activity and improved physical fitness is proven to be an effective way to reduce mortality (1-4). In addition to reduced mortality physical activity improves health (5) and reduces the risk of many diseases such as cardiovascular disease (6), type 2 diabetes (7), osteoporosis (8), obesity (9), depression (10) and breast - and rectal cancer (11). Maximal oxygen uptake (VO2max) is shown to be the strongest prognostic predictor of mortality in both healthy and in patients with cardiovascular disease (12-15). Aerobic interval training with high intensity (90-95 % of maximum heart rate) has proven very effective for increasing VO2max, and more effective than exercise with moderate intensity. Studies with this type of training have been conducted in our research group of healthy (16), and in patients with coronary artery disease (17), heart failure (18), intermittent claudication (19) and metabolic syndrome (20). An interesting point in rehabilitation purposes is how quickly it is possible to increase VO2max and thus better health. More studies are therefore needed to increase knowledge about the mechanisms and timing behind the physical adaptation aerobic interval training provides and how training adaptations are lost during detraining. We will therefore conduct a training study with interval training on healthy young people with a view to clarifying these matters.

**Specific objectives of the study**

The study's overall objective is to contribute to increased knowledge of effective fitness training for healthy patients and athletes, and thereby motivate lifelong healthy exercise. Primary outcome measures are VO2max.

*The project's main objective is divided into three:*

1) Examine the 24 interval sessions conducted over a shorter period (8 sessions per week for 3 weeks) may increase VO2max corresponding to the same number of sessions conducted over a " normal" amount of time (3 sessions per week for 8 weeks).

2) Determine how quickly VO2max decreases after exercise stops.

3) Describe how selected physiological parameters are influenced by the two training regimes.

**Methods**

*Inclusion criteria*

24 people randomized to two groups, both sexes included . Inclusion criteria will be: Age between 18-27 years, BMI < 27.5, not smoking , exercising less than 1-2 conditioning sessions per week, VO2max < 60 for men and < 50 for women. Exclusion criteria will be hypertension, diabetes and congenital heart disease.

*Number of subjects*

With an expected increase of oxygen uptake in 5 ml • kg - 1 • min- 1 , the standard deviation of 4 , and a p- value of 0.05 to 12 volunteers provide a statistical power of 83%. A total of 24 subjects will therefore be randomized and placed in two different training groups.

*randomization*

Randomization performed automatically by the device for Applied Clinical Research, Faculty of Medicine, NTNU.

*Testing*

All subjects tested for VO2max, cardiac output, muscle biopsies of skeletal muscle (rectus femoris), arterialdiameter , echocardiography of the heart, blood tests , blood pressure , diffusion capacity of the lung membrane. All tests carried out before and after the training. Only condition is tested after every 8 workout.

Testers know which group the participant belongs because they also account for the continual monitoring. Results achieved investigated as little as possible while the study is ongoing. It's very clear criteria for determination of maximal oxygen uptake followed punctual (21).

VO2max: First performed 10 minutes warm up mask attached and measurements started. Incline of the treadmill set at 10 % and the rate bull by 1 km / h every minute to exhaustion. For statistics of oxygen uptake at multiple time points used mixed linear model.

Vessel diameter: Diameter of brachial artery measured by ultrasound on the subject present. The artery measured at rest and after reactive hyperemia.

Echocardiography: Full echocardiography including tissue Doppler recordings performed on subjects lying at rest and during submaximal work load on the bicycle.

Blood tests: Blood analysis is taken for determination of blood count, lipid profile

Lungdiffusjon: Diffusjonstest which provides a measure of the diffusion of oxygen from the lungs to the bloodstream. Measured by " CO - diffusion single breath method . " The study consists of a gas mixture consisting of 10 % helium, 0.3 % CO, 21% oxygen and the rest nitrogen inhale slowly from a maximum exhalation. The breath is then held for 10 seconds and quiet exhalation. A maximum of 4 trials done in a series. This is also a standard pulmonary function tests being done daily in the routine diagnosis of pulmonary clinic at St. Olav's Hospital (approximately 1,200 annually) and other hospitals in the region. The survey has no contraindications and gives no increase of CO in the blood of those surveyed. The method presents no discomfort to the subjects.

Muscle biopsy: biopsy performed by the participant under local anesthesia followed by an average of about 0.5 centimeters through the skin. Then, one biopsy needle inserted into the muscle and two small samples (15-30 mg) is removed.

Exercise Intervention:

The project is divided into two phases. All training takes place as interval training is run on the treadmill at work physiological laboratory of medical research, NTNU. Hatle and Støbakk is responsible for training and record attendance. It is not permitted in some exercise while the study is ongoing.

Phase 1:

Group A carries out 24 sessions over 8 weeks (3 sessions per week). Starting September 29, 2008. Training takes place Monday, Wednesday and Friday. Testing of VO2max performed every 8 work by individual mask applied during the first interval draw.

Group B carries out 24 sessions over 3 weeks (8 sessions per week). Same type of training group A. Group B starts training when team A has completed its 4th week (27 October 2008). Training takes place as one session Monday, 2 Tuesday 1 Wednesday 2 Thursday, one Friday and one Saturday. Testing of VO2max performed every 8 work by individual mask applied during the first interval draw.

Phase 2:

After completing training in Phase 1, cease all training (detraining). This period is going to last for 9 weeks including Christmas holiday. The phase starts immediately after Phase 1 is completed, ie group B starts a week before detraining group A. Testing of VO2max conducted biweekly.

*Feasibility*

The training program conceivably feasible because the same is done on patients groups at our University earlier. The criteria underlying the termination of the study is that muscle fatigue occurs before the desired target level occurs. However, each case is considered individually since we believe training is feasible without dropping. There is no obvious reason why the task will not be completed in a timely manner.

Schedule

Recruitment of participants. Start of pre - testing in September 2008.

*Group A:*

Phase 1 : Starting 29 September 2008 , eight weeks to 24 November 2008.

Phase 2: Starting 24 November 2008 to 19 January 2009.

*Group B:*

Phase 1 : Starting 27 October. 3 weeks to 17 November 2008.

Phase 2: Starting 17 November 2008 and 12 January 2009.

*Any ethical aspects of the study*

It should in this study are trained fairly hard and the risk of overloading the participants present. All training shall be carried out at run uphill on the treadmill which reduces the load they are exposed. The muscle biopsy will be taken out a small muscle sample from the thigh. This is done without pain for the participants but some may feel that they farm in muscle 2-3 days after the sample is taken. Figures from the study kept anonymous for five years under the current rules.

It is applied to the national authorithies for creating Biobank in connection with the study. These are granted.

**Publication plan**

The study will be published in international journals.

**Employees**

Ulrik Wisløff, professor, NTNU, Faculty of Medicine

Øivind Rognmo, postdoc, NTNU, Faculty of Medicine

Arnt Erik Tjønna, postdoc, NTNU, Faculty of Medicine

Håvard Hatle, medical student, NTNU, Faculty of Medicine

Per Kristian Støbakk, medical student, NTNU, Faculty of Medicine

Carlotte Bjørk Ingul, MD, postdoc, NTNU, Faculty of Medicine

Sigurd Steinshamn, MD, professor, NTNU, Faculty of Medicine

Eirik Skogvoll, MD, professor, NTNU, Faculty of Medicine

Eivind Brønstad, MD, PhD, NTNU, Faculty of Medicine

Harald Edvard Mølmen, MD, PhD NTNU, Faculty of Medicine

# References

1. Blair SN, Kohl HW, 3rd, Paffenbarger RS, Jr., Clark DG, Cooper KH, Gibbons LW. Physical fitness and all-cause mortality. A prospective study of healthy men and women. Jama 1989;262:2395-401.

2. Sandvik L, Erikssen J, Thaulow E, Erikssen G, Mundal R, Rodahl K. Physical fitness as a predictor of mortality among healthy, middle-aged Norwegian men. N Engl J Med 1993;328:533-7.

3. Paffenbarger RS, Jr., Hyde RT, Wing AL, Hsieh CC. Physical activity, all-cause mortality, and longevity of college alumni. N Engl J Med 1986;314:605-13.

4. Vanhees L, Fagard R, Thijs L, Staessen J, Amery A. Prognostic significance of peak exercise capacity in patients with coronary artery disease. J Am Coll Cardiol 1994;23:358-63.

5. Booth FW, Chakravarthy MV, Gordon SE, Spangenburg EE. Waging war on physical inactivity: using modern molecular ammunition against an ancient enemy. J Appl Physiol 2002;93:3-30.

6. Thompson PD, Buchner D, Pina IL, et al. Exercise and physical activity in the prevention and treatment of atherosclerotic cardiovascular disease: a statement from the Council on Clinical Cardiology (Subcommittee on Exercise, Rehabilitation, and Prevention) and the Council on Nutrition, Physical Activity, and Metabolism (Subcommittee on Physical Activity). Circulation 2003;107:3109-16.

7. Albright A, Franz M, Hornsby G, et al. American College of Sports Medicine position stand. Exercise and type 2 diabetes. Med Sci Sports Exerc 2000;32:1345-60.

8. Vuori IM. Dose-response of physical activity and low back pain, osteoarthritis, and osteoporosis. Med Sci Sports Exerc 2001;33:S551-86; discussion 609-10.

9. Ross R, Janssen I. Physical activity, total and regional obesity: dose-response considerations. Med Sci Sports Exerc 2001;33:S521-7; discussion S528-9.

10. Pollock KM. Exercise in treating depression: broadening the psychotherapist's role. J Clin Psychol 2001;57:1289-300.

11. Lee IM. Physical activity and cancer prevention--data from epidemiologic studies. Med Sci Sports Exerc 2003;35:1823-7.

12. Myers J, Prakash M, Froelicher V, Do D, Partington S, Atwood JE. Exercise capacity and mortality among men referred for exercise testing. N Engl J Med 2002;346:793-801.

13. Gulati M, Pandey DK, Arnsdorf MF, et al. Exercise capacity and the risk of death in women: the St James Women Take Heart Project. Circulation 2003;108:1554-9.

14. Kavanagh T, Mertens DJ, Hamm LF, et al. Prediction of long-term prognosis in 12 169 men referred for cardiac rehabilitation. Circulation 2002;106:666-71.

15. Kavanagh T, Mertens DJ, Hamm LF, et al. Peak oxygen intake and cardiac mortality in women referred for cardiac rehabilitation. J Am Coll Cardiol 2003;42:2139-43.

16. Helgerud J, Hoydal K, Wang E, et al. Aerobic high-intensity intervals improve VO2max more than moderate training. Med Sci Sports Exerc 2007;39:665-71.

17. Rognmo O, Hetland E, Helgerud J, Hoff J, Slordahl SA. High intensity aerobic interval exercise is superior to moderate intensity exercise for increasing aerobic capacity in patients with coronary artery disease. Eur J Cardiovasc Prev Rehabil 2004;11:216-22.

18. Wisloff U, Stoylen A, Loennechen JP, et al. Superior cardiovascular effect of aerobic interval training versus moderate continuous training in heart failure patients: a randomized study. Circulation 2007;115:3086-94.

19. Slordahl SA, Wang E, Hoff J, Kemi OJ, Amundsen BH, Helgerud J. Effective training for patients with intermittent claudication. Scand Cardiovasc J 2005;39:244-9.

20. Tjonna AE, Lee SJ, Rognmo O, et al. Aerobic interval training versus continuous moderate exercise as a treatment for the metabolic syndrome: a pilot study. Circulation 2008;118:346-54.

21. Rognmo et al. Endothelial function in highly endurance-trained men: effects
of acute exercise./
/J Strength Cond Res. 2008 Mar;22(2):535-42.
